# Association of waist-to-hip ratio with risk of kidney stones in hypertensive population: a population-based cross-sectional study

**DOI:** 10.3389/fendo.2025.1448137

**Published:** 2025-09-23

**Authors:** Feiyan Zhang, Jie Lv, Maidinaimu Aibibula, Jiahui Li, Xuechao Ma, Yutong He, Haiyang Zhou

**Affiliations:** ^1^ Institute of Medical Sciences, General Hospital of Ningxia Medical University, Yinchuan, Ningxia, China; ^2^ Clinical Laboratory Center, Tumor Hospital Affiliated to Xinjiang Medical University, Urumqi, China; ^3^ Department of Pathology and Pathophysiology, Ningxia Medical University, Yinchuan, Ningxia, China

**Keywords:** National Health And Nutrition Examination Survey, waist-to-hip ratio, kidney stones, hypertension, cross-sectional

## Abstract

**Background:**

Kidney stones (KS) are a prevalent urological condition with high recurrence rates and substantial treatment costs. Hypertension has been identified as an independent risk factor for KS. The waist-to-hip ratio (WHR) has also been associated with KS; however, its relationship with KS in the hypertensive population remains unexplored. Through a population-based cross-sectional study, this study aimed to assess the association between WHR and KS risk in the hypertension population.

**Methods:**

Data from the National Health and Nutrition Examination Survey (NHANES) 2007 to 2020 were analyzed, comprising 1,572 hypertensive patients. Logistic regression and restricted cubic splines analysis were performed to examine the association between WHR and KS. Receiver operator characteristic (ROC) analyses were performed to assess the diagnostic ability of several human obesity-related indices for KS.

**Results:**

A total of 1,572 hypertensive patients were included in the final study, with a mean age of 54.87 years and a prevalence of KS of 12.28%. The study found a significant association between WHR and KS, even after adjusting for confounding factors, with higher WHR associated with a higher likelihood of KS occurrence (OR = 1.63, 95%CI: 1.13, 2.34). This positive association is linear (non-linear *p >*0.05). ROC curve results showed that WHR had the highest AUC. Subgroup analysis showed consistent associations in almost all populations, with no significant interaction effects (*p* for interaction > 0.05). Finally, sensitivity analysis further confirmed the stability of the results.

**Conclusion:**

In the hypertensive population, WHR is positively associated with the likelihood of KS. These findings highlight the importance of considering WHR as a risk factor for KS in hypertensive individuals, providing valuable insights for managing KS in this population.

## Introduction

Kidney stones (KS) are a common urological condition globally, characterized by the accumulation of inorganic and organic substances within the renal parenchyma (1). In recent years, the prevalence of KS has been on the rise, affecting approximately 13% of the population in the Northern Hemisphere and reaching up to 19.1% in specific regions of Asia (2). Notably, KS not only occurs frequently but also has a significant tendency to recur, with nearly half of affected individuals likely to experience another episode during their lifetime (3). Moreover, the economic burden associated with KS is substantial. In the United States alone, the annual cost of treating KS exceeded 5 billion dollars in 2005, and projections indicate a further increase of 1.24 billion dollars by 2030 (4, 5). Multiple factors influence the development of KS, among which hypertension stands out as a critical independent risk factor. Historical research has shown that middle-aged men with hypertension are at a 96% increased risk of developing KS, highlighting the significant impact of elevated blood pressure on renal stone formation (6). Recent cross-sectional studies from Iran have provided further evidence, confirming hypertension as an independent risk factor for KS development (7). Given the rapid increase in the number of individuals with hypertension in recent years, it is imperative to focus on the risk factors associated with KS development in this population (8).

Previous research has highlighted the association between waist-to-hip ratio (WHR) and the risk of KS. A study utilizing data from the UK Biobank revealed that WHR, independent of body mass index (BMI), increased the risk of KS by 24% (9). Another prospective study conducted in Taiwan, involving 25,268 participants with an average follow-up time of five months, reported an independent association between WHR and the risk of KS (10). These findings underscore the significant role of WHR in the development of KS.

Hypertension and central obesity (as measured by WHR) are both important components of the metabolic syndrome, which is strongly associated with an increased risk of KS (11, 12). Hypertension and central obesity often coexist and may have synergistic effects on metabolic disorders that are known to increase the risk of stone formation (13, 14). Although previous studies have explored the relationship between hypertension and KS, as well as the independent relationship between WHR and KS, the specific interactions between WHR and KS in hypertensive populations remain less well-studied. Given that hypertension is a prevalent condition with significant health implications, understanding the role of WHR in this specific population may provide targeted insights for prevention strategies (15).

The objective of this research is to assess the association between WHR and the propensity for KS in hypertensive individuals, utilizing data from the National Health and Nutrition Examination Survey (NHANES). This study seeks to furnish valuable insights for this particular population, thereby contributing significantly to the preventative strategies against KS development in those affected by hypertension.

## Methods

### Study population

**Figure 1:**
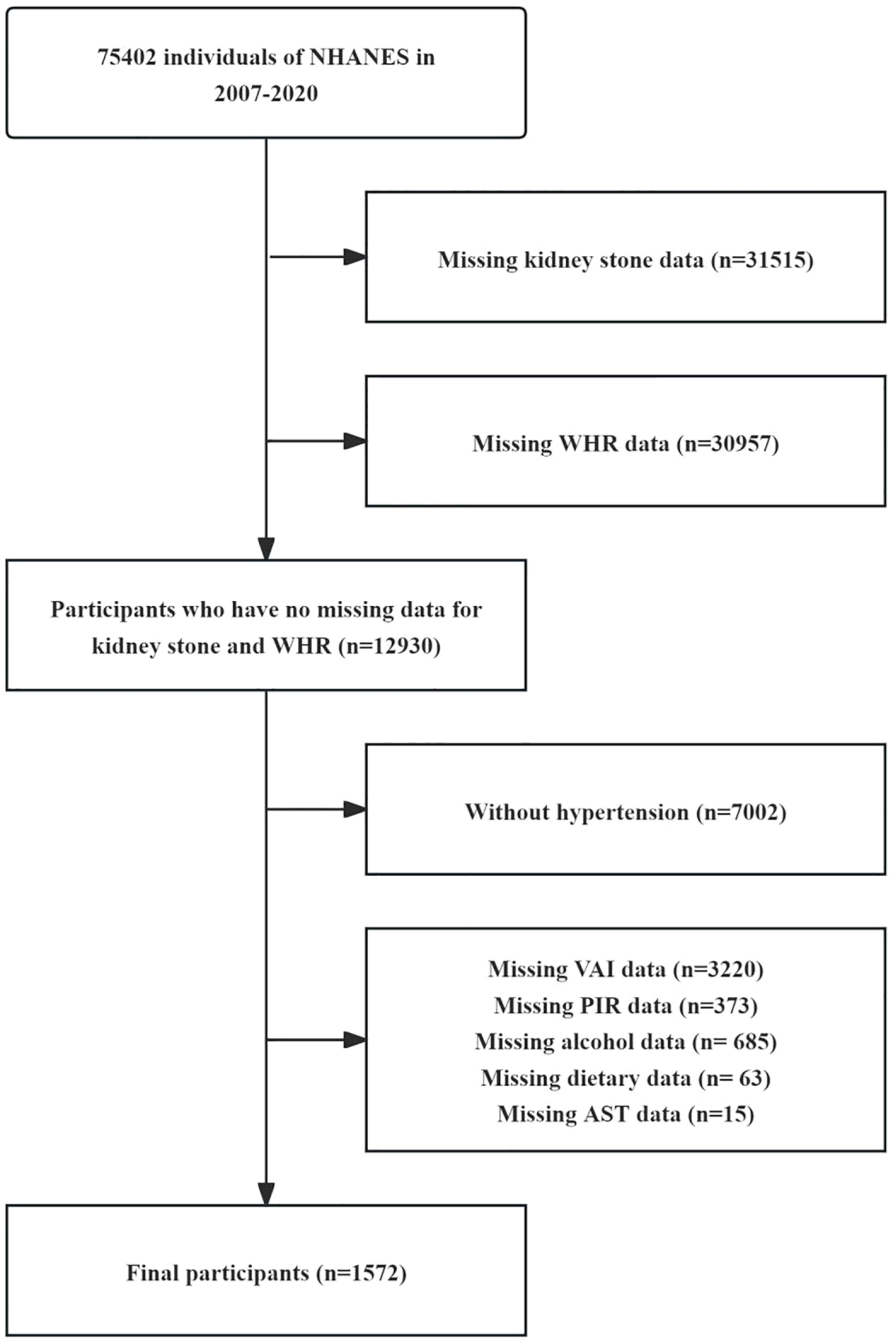
Flow chart of the study subjects.

This study utilizes data from the NHANES database, which is a comprehensive public health monitoring project conducted in the United States. A primary objective of NHANES is to assess the health and nutritional status of Americans. The data used in our study were collected from seven cycles of NHANES between 2007 and 2020. The total sample size consisted of 75,402 participants included in the analysis. In the selection process for this study, after excluding 31,515 participants who lacked information on KS status, 30957 participants who lacked information on WHR, 7,002 participants who did not have hypertension, 4,356 participants who lacked information on other covariates, the final study included 1,572 patients and was divided into four quartile groups according to their WHR. The entire selection process is depicted in **Figure 1**.

### Primary variables

The KS patients are those who answered “yes” to the question “Have you ever had kidney stones?”. Self-reported hypertension or blood pressure exceeding 140 mmHg for a systolic reading, 90 mmHg for a diastolic reading, or the use of antihypertensive medications are considered hypertension.

The formulas for calculating WHR, BMI, VAI, and TyG are outlined below (16–18):


$$\text{WHR}=\frac{\text{WC }\left(\text{cm}\right)}{\text{Hip circumference }\left(\text{cm}\right)}$$

$$\text{BMI}=\frac{\text{weight }\left(\text{kg}\right)}{{\text{height}}^{2}\text{(m})}$$

$${\text{VAI}}_{\text{male}}\text{=[}\frac{\text{WC (cm)}}{39.68 + 1{.88*\text{BMI (kg/m}}^{2})}]*[\frac{\text{triglycerides (mmol/L)}}{1.03}]*[\frac{1.31}{\text{HDL(mmol/L)}}]$$

$${\text{VAI}}_{\text{female}}\text{=[}\frac{\text{WC (cm)}}{36.58\;+\;(1.89\;\times \;{\text{BMI (kg/m}}^{2})}]*[\frac{\text{triglycerides (mmol/L)}}{0.81}]*[\frac{1.52}{\text{HDL (mmol/L}}]$$

$$\text{TyG}=\text{Ln}[\frac{\text{triglycerides}\;(\text{mg}/\text{dL})\;*\text{ glucose}\;(\text{mg}/\text{dL})}{2}]$$

### Covariates

The study analyzed demographic, laboratory, physical examination, and questionnaire data from the NHANES database based on historical literature and clinical experience (19, 20). Continuous variables include age, poverty income ratio (PIR), BMI, level of glucose, alanine aminotransferase (ALT), aspartate transaminase (AST), uric acid, calcium, dietary energy, dietary protein, and estimated glomerular filtration rate (eGFR). Sex, race, smoking, drinking, physical activity, history of type 2 diabetes mellitus (T2DM), and hyperlipidemia are considered categorical variables. Races are categorized as Mexican American, Non-Hispanic Black, Non-Hispanic White, Other Hispanic, and Other Race. Smoking is classified as never, former, or now. Drinking is categorized according to drinking habits: non-drinkers (never drank or quit in the past year), moderate drinkers (< 2 cups/day for men and< 1 cup/day for women), or heavy drinkers (≥ 2 cups/day for men and ≥ 1 cup/day for women) (21). The diagnosis of T2DM was based on the following criteria: (1) fasting plasma glucose of 126 mg/dL; (2) 2-hour oral glucose tolerance test of 200 mg/dL; (3) HbA1c of 6.5%; or (4) diagnosis by a physician or medication use. Adult Treatment Panel III of the National Cholesterol Education Program classified hyperlipidemia as total cholesterol 200 mg/dL, triglycerides 150 mg/dL, HDL 40 mg/dL in males and 50 mg/dL in females, or low-density lipoprotein 130 mg/dL, and persons who reported using cholesterol-lowering drugs were also classified as having hyperlipidemia (22). Exercise intensity is usually expressed by the metabolic equivalent of task (MET), which refers to the ratio of energy consumed during PA to energy consumed at rest (23). One MET is equivalent to the resting metabolic rate or energy expenditure when awake and sitting quietly. Moderate PA has a MET value of 3 to 5.9 MET; vigorous PA has a MET value of 6 or higher (24).

### Statistical analysis

We categorized the participants into four groups according to the quartiles of WHR. The differences between groups were compared using t-tests or one-way ANOVA for continuous variables. Chi-square tests were used to assess group differences for categorical variables presented as frequencies and percentages. We used univariate and multivariate logistic regression models to examine the associations between WHR and KS. Three models were employed in the analysis: Crude model (unadjusted), Model 1 (adjusted for age, sex, race, and PIR), and Model 2 (additional adjustment for BMI, smoking, drinking, T2DM, glucose, uric acid, calcium, ALT, AST, dietary energy, dietary protein, eGFR, physical activity, and hyperlipidemia). Restricted cubic splines (RCS), adjusted for confounding factors, were used to visualize the association between WHR and the prevalence of KS. To compare the ability of obesity and lipid-related indices to diagnose KS, the area under the receiver operating characteristic curve (ROC) was used to assess the ability of anthropometric indices to diagnose KS. Finally, subgroup analyses were performed according to age, gender, ethnicity, BMI, lifestyle habits (smoking and drinking), presence of T2DM, and the presence of metabolic abnormalities (abnormalities of hepatic metabolism defined as ALT or AST >40 u/L, uric acid metabolism defined as uric acid metabolism >7 mg/dL in men and >6 mg/dL in women, and calcium metabolism defined as calcium metabolism<2.25 mmol/L and >2.65 mmol/L) order to study the association between WHR and KS in hypertensive patients with different characteristic (25–27). Sensitivity analyses (1) further adjusted the NHANES survey period according to Model 2, and (2) excluded individuals with abnormal renal function (eGFR<60), verifying the stability of the results.

All statistics were calculated using weighted data from the NHANES database, and the weights were chosen based on the ratio of the weights of the samples examined in the laboratory to the number of cycles. Analyzed using R software (version 4.2.1), two-tailed *p* values less than 0.05 were used to determine statistical significance.

## Results

### Baseline characteristics

A total of 1,572 hypertensive subjects with a mean age of 54.87 years were included in this study, of whom 45.92% were females. The average WHR of the participants was 11.64. **Table 1** shows the clinical characteristics of the participants based on KS grouping. There were significant statistical differences between participants with and without KS in terms of AST, WHR, race, and smoking (*p*<0.05). Compared to non-KS patients, KS patients had lower levels of AST, higher WHR, and a higher proportion of Other Hispanics and smokers.

**Table 1:** Characteristics of the hypertensive population based on the presence of KS.

Characteristics	Overall (N = 1572)	No-KS (N = 1391)	With-KS (N = 181)	*p* value
Age, years	54.87(0.77)	54.99(0.75)	54.01(1.76)	0.541
Sex, n (%)				0.070
Female	738(45.92)	668(47.38)	70(35.46)	
Male	834(54.08)	723(52.62)	111(64.54)	
Race, n (%)				0.005
Mexican American	147(5.63)	129(5.52)	18(6.35)	
Non-Hispanic Black	469(12.14)	436(12.87)	33(6.91)	
Non-Hispanic White	580(67.33)	504(67.25)	76(67.86)	
Other Hispanic	148(6.24)	121(5.56)	27(11.16)	
Other Race	228(8.67)	201(8.80)	27(7.71)	
PIR	3.25(0.08)	3.24(0.08)	3.39(0.16)	0.396
BMI, kg/m^2^	31.48(0.30)	31.53(0.32)	31.11(0.45)	0.376
Glucose, mg/dL	117.59(1.78)	118.16(2.03)	113.51(2.32)	0.168
ALT, U/L	26.32(1.13)	26.79(1.23)	22.94(1.52)	0.036
AST, U/L	24.12(0.81)	24.52(0.91)	21.30(0.70)	0.007
Uric acid, mg/dL	5.78(0.07)	5.75(0.07)	6.04(0.21)	0.162
Calcium, mmol/L	2.32(0.01)	2.33(0.01)	2.32(0.01)	0.410
WC, cm	106.04(0.74)	105.90(0.77)	107.04(1.00)	0.216
WHR	11.64(0.04)	11.60(0.04)	11.90(0.08)	0.001
VAI	1.87(0.08)	1.85(0.08)	2.09(0.18)	0.225
TyG	8.68(0.04)	8.68(0.04)	8.69(0.05)	0.845
eGFR, mL/min/1.73m²	88.61(0.95)	88.54(0.96)	89.11(2.61)	0.833
Dietary energy, kcal	2209.19(37.90)	2204.78(31.73)	2240.65(174.55)	0.836
Dietary protein, g	83.33(1.57)	83.42(1.43)	82.68(5.31)	0.885
T2DM, n (%)				0.704
No	1073(74.07)	958(73.84)	115(75.71)	
Yes	499(25.93)	433(26.16)	66(24.29)	
Smoking, n (%)				< 0.001
Never	821(54.45)	727(57.06)	94(35.85)	
Former	436(28.85)	381(25.80)	55(50.64)	
Now	315(16.70)	283(17.15)	32(13.51)	
Drinking, n (%)				0.669
Never	171(8.49)	137(8.23)	34(10.37)	
Moderate	1058(68.72)	947(68.77)	111(68.38)	
Heavy	343(22.79)	307(23.00)	36(21.25)	
Hyperlipidemia, n (%)				0.725
No	337(21.69)	302(21.51)	35(23.01)	
Yes	1235(78.31)	1089(78.49)	146(76.99)	
Physical activity, n (%)				0.858
No	1050(62.49)	931(62.34)	119(63.59)	
Moderate	439(31.36)	388(31.26)	51(32.09)	
Vigorous	83(6.15)	72(6.40)	11(4.32)	

Abbreviations: BMI, body mass index; PIR, poverty income ratio; ALT, alanine aminotransferase; AST, aspartate transaminase; WC, waist circumference; T2DM, type 2 diabetes mellitus; WHR, waist-to-hip ratio; VAI, visceral adiposity index; TyG, triglyceride glucose; eGFR, estimated glomerular filtration rate.

All values are expressed as a proportion (%) or mean ± standard error.

**Table 2** provides further stratification of clinical characteristics based on WHR quartiles. WHR quartiles showed statistical significance in age, sex, race, BMI, glucose, ALT, AST, uric acid, WC, VAI, TYG, eGFR, dietary energy, dietary protein, T2DM, KS, hyperlipidemia, physical activity, and smoking (*p*<0.05). The highest WHR quartile was older, more male, and non-Hispanic white, had higher BMI, glucose, ALT, AST, uric acid, WC, VAI, TyG, eGFR, dietary energy, dietary protein levels, and higher rates of comorbid T2DM, KS, and hyperlipidemia, and lived their lives with low physical activity and smoking.

**Table 2:** Characteristics of the study population by WHR quartiles.

Characteristics	Total (N = 1572)	Q1 (N = 395)	Q2 (N = 391)	Q3 (N = 393)	Q4 (N = 393)	*p* value
Age, years	54.87(0.77)	54.39(0.88)	52.60(1.66)	57.13(1.04)	55.90(1.16)	0.029
Sex, n (%)						<0.001
Female	738(45.92)	310(80.09)	233(51.94)	144(33.49)	51(11.57)	
Male	834(54.08)	85(19.91)	158(48.06)	249(66.51)	342(88.43)	
Race, n (%)						0.029
Mexican American	147(5.63)	24(3.58)	31(4.75)	45(7.55)	47(7.22)	
Non-Hispanic Black	469(12.14)	136(13.85)	127(12.35)	115(13.52)	91(8.78)	
Non-Hispanic White	580(67.33)	145(68.06)	124(65.40)	136(63.75)	175(71.65)	
Other Hispanic	148(6.24)	36(5.61)	27(5.18)	45(8.60)	40(6.06)	
Other Race	228(8.67)	54(8.90)	82(12.32)	52(6.58)	40(6.30)	
PIR	3.25(0.08)	3.31(0.16)	3.31(0.10)	3.31(0.14)	3.08(0.14)	0.221
BMI, kg/m^2^	31.48(0.30)	28.97(0.60)	31.87(0.40)	31.69(0.45)	33.72(0.50)	<0.001
Glucose, mg/dL	117.59(1.78)	106.42(1.20)	120.81(4.54)	118.36(1.94)	126.11(3.04)	<0.001
ALT, U/L	26.32(1.13)	22.98(2.33)	23.71(0.80)	25.66(0.84)	33.44(2.38)	<0.001
AST, U/L	24.12(0.81)	24.82(1.69)	22.17(0.63)	22.31(0.33)	26.99(1.57)	0.027
Uric acid, mg/dL	5.78(0.07)	5.16(0.11)	5.88(0.11)	6.14(0.11)	6.08(0.13)	<0.001
Calcium, mmol/L	2.32(0.01)	2.33(0.01)	2.32(0.01)	2.32(0.01)	2.32(0.01)	0.499
WC, cm	106.04(0.74)	94.81(1.12)	105.29(0.91)	108.96(0.97)	117.03(1.08)	<0.001
VAI	1.87(0.08)	1.40(0.09)	1.77(0.09)	2.17(0.08)	2.27(0.15)	<0.001
TyG	8.68(0.04)	8.36(0.05)	8.68(0.05)	8.84(0.03)	8.89(0.06)	<0.001
eGFR, mL/min/1.73m²	88.61(0.95)	88.54(1.04)	91.91(1.65)	85.74(1.72)	87.65(1.49)	0.014
Dietary energy, kcal	2209.19(37.90)	1934.14(65.32)	2094.68(54.20)	2407.51(73.47)	2471.43(105.21)	<0.001
Dietary protein, g	83.33(1.57)	73.12(2.49)	79.04(2.43)	88.54(2.12)	94.95(4.04)	<0.001
T2DM, n (%)						0.004
No	1073(74.07)	325(87.32)	278(73.00)	256(67.92)	214(65.53)	
Yes	499(25.93)	70(12.68)	113(27.00)	137(32.08)	179(34.47)	
Smoking, n (%)						<0.001
Never	821(54.45)	253(67.72)	213(55.26)	191(50.75)	164(41.79)	
Former	436(28.85)	77(13.58)	99(28.90)	119(35.15)	141(40.61)	
Now	315(16.70)	65(18.71)	79(15.84)	83(14.10)	88(17.59)	
Drinking, n (%)						0.236
Never	171(8.49)	51(6.47)	45(10.67)	47(9.11)	28(7.92)	
Moderate	1058(68.72)	274(68.16)	264(71.72)	249(62.36)	271(71.63)	
Heavy	343(22.79)	70(25.37)	82(17.61)	97(28.52)	94(20.45)	
KS, n (%)						0.021
No	1391(87.72)	364(92.00)	355(90.05)	341(86.66)	331(81.31)	
Yes	181(12.28)	31(8.00)	36(9.95)	52(13.34)	62(18.69)	
Hyperlipidemia, n (%)						0.028
No	337(21.69)	110(29.28)	81(18.80)	70(16.90)	76(20.34)	
Yes	1235(78.31)	285(70.72)	310(81.20)	323(83.10)	317(79.66)	
Physical activity, n (%)						0.019
No	1050(62.49)	255(61)	270(70.66)	241(53.11)	284(63.52)	
Moderate	439(31.36)	112(30.56)	108(25.05)	126(40.04)	93(31.53)	
Vigorous	83(6.15)	28(8.43)	13(4.29)	26(6.85)	16(4.95)	

Abbreviations: BMI, body mass index; PIR, poverty income ratio; ALT, alanine aminotransferase; AST, aspartate transaminase; WC, waist circumference; T2DM, type 2 diabetes mellitus; WHR, waist-to-hip ratio; VAI, visceral adiposity index; TyG, triglyceride glucose; eGFR, estimated glomerular filtration rate.

WHR quartiles: 11.06≤Q1, 11.06<Q2 ≤ 11.72, 11.72<Q3 ≤ 12.29, and 12.93<Q4.

All values are expressed as a proportion (%) or mean ± standard error.

### The association between WHR and KS in hypertensive individuals

In **Table 3**, we analyze the association between WHR and KS in hypertensive patients by logistic regression. After adjusting for all confounding factors, Model 2 demonstrates a significant association between WHR and the prevalence rate of KS (OR = 1.63, 95% CI: 1.13, 2.34). Each 1 standard deviation (SD) increase in WHR is associated with a 63% increase in the likelihood of KS in hypertensive individuals. Furthermore, when analyzing the quartiles of WHR, participants in Q4 have a 3.45 times higher likelihood of KS compared to those in Q1 (OR = 3.45, 95% CI: 1.32, 9.02). The association between different WHR quartiles and the occurrence of KS exhibited heterogeneity (*p* for trend = 0.009).

**Table 3:** Association between WHR and KS in hypertensive patients.

Parameter	Crude model		Model 1		Model 2	
	OR (95%CI)	*p*	OR (95%CI)	*p*	OR (95%CI)	*p*
WHR^a^	1.41(1.14, 1.75)	0.002	1.40(1.04, 1.88)	0.030	1.63(1.13, 2.34)	0.013
WHR quartiles						
Q1	ref		ref		ref	
Q2	1.27(0.68, 2.39)	0.448	1.23(0.69, 2.19)	0.463	1.08(0.46, 2.51)	0.849
Q3	1.77(0.92, 3.40)	0.084	1.72(0.89, 3.31)	0.102	1.57(0.87, 2.83)	0.122
Q4	2.64(1.32, 5.28)	0.007	2.61(1.12, 6.10)	0.028	3.45(1.32, 9.02)	0.016
*p* for trend		0.004		0.029		0.009

a: 1 standard deviation (SD) WHR was established in association with KS.

Crude model: no adjusted;

Model 1: adjusted for age, sex, race, and PIR;

Model 2: adjusted for age, sex, race, PIR, BMI, smoking, drinking, T2DM, glucose, uric acid, calcium, ALT, AST, dietary energy, dietary protein, eGFR, physical activity, and hyperlipidemia.

### Dose-response relationship

**Figure 2:**
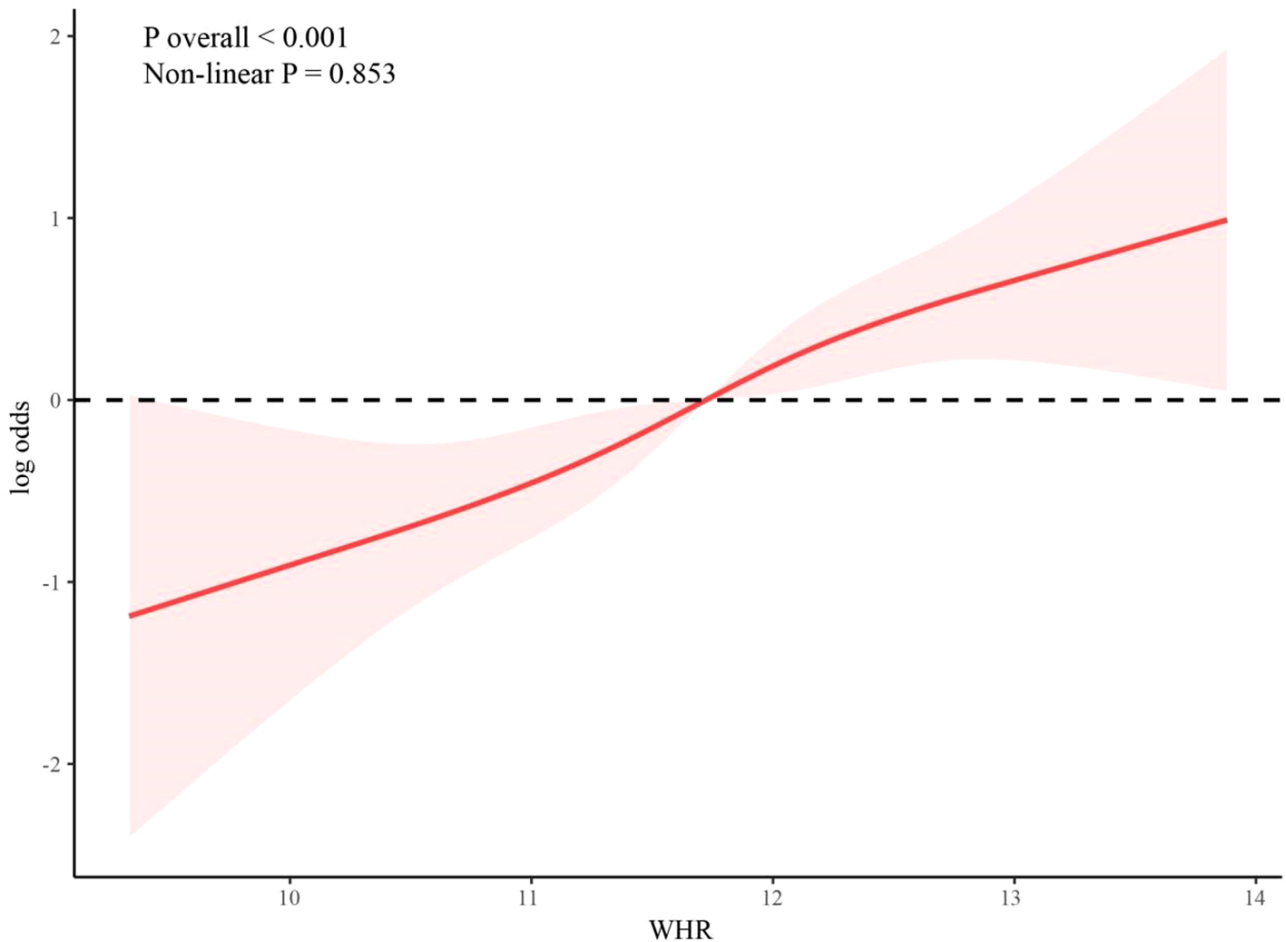
Dose-response association between WHR and KS in hypertensive patients.

RCS is used in **Figure 2** to explore the association between WHR and KS among hypertensive individuals. After adjusting for age, sex, race, PIR, BMI, smoking, drinking, T2DM, glucose, uric acid, calcium, ALT, AST, dietary energy, dietary protein, eGFR, physical activity, and hyperlipidemia, the association between WHR and KS remained statistically significant (*p* overall<0.001). The positive association between WHR and KS is linear (non-linear *p* = 0.853).

### Diagnostic efficacy of various obesity-related indicators for KS in hypertensive patients

**Figure 3:**
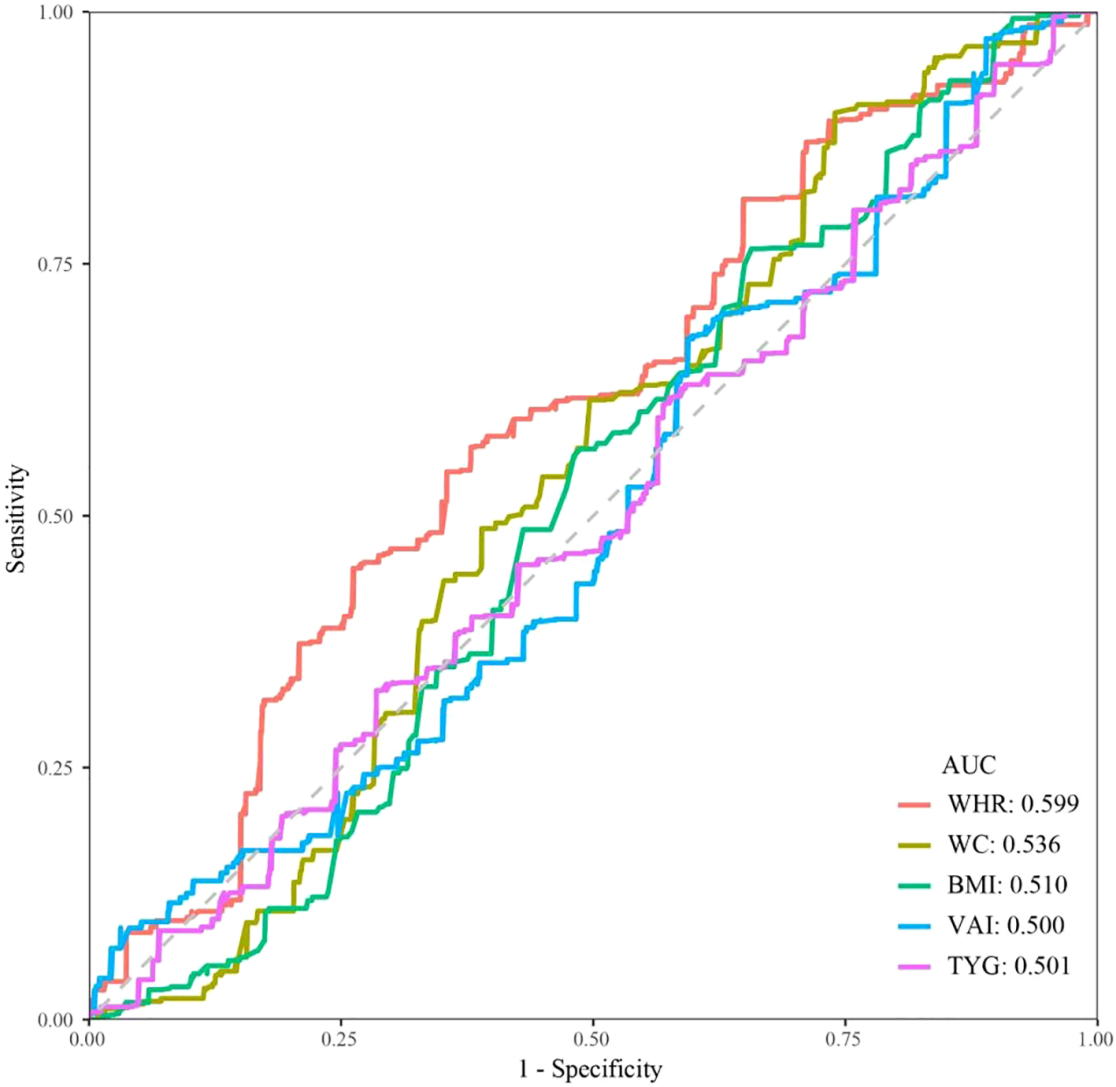
ROC curves and AUC values of five obesity-related indicators (WHR, WC, BMI, VAI, and TyG) for predicting KS in hypertensive patients.

We explored the association of the remaining obesity-related indicators (WC, BMI, VAI, and TyG) except for WHR, with KS in hypertensive patients by multivariate logistic regression. The results suggest that all of these indicators are associated with an increased risk of KS (**Supplementary Table S1**). **Figure 3** shows the ROC curves of various obesity-related indices for the diagnosis of KS. WHR showed the highest discriminatory power in predicting the development of KS in hypertensive patients (AUC = 0.599), followed by WC (AUC = 0.536). In contrast, BMI, VAI, and TyG had lower predictive accuracy with AUCs of 0.510, 0.500, and 0.518, respectively.

### Subgroup analysis

**Figure 4:**
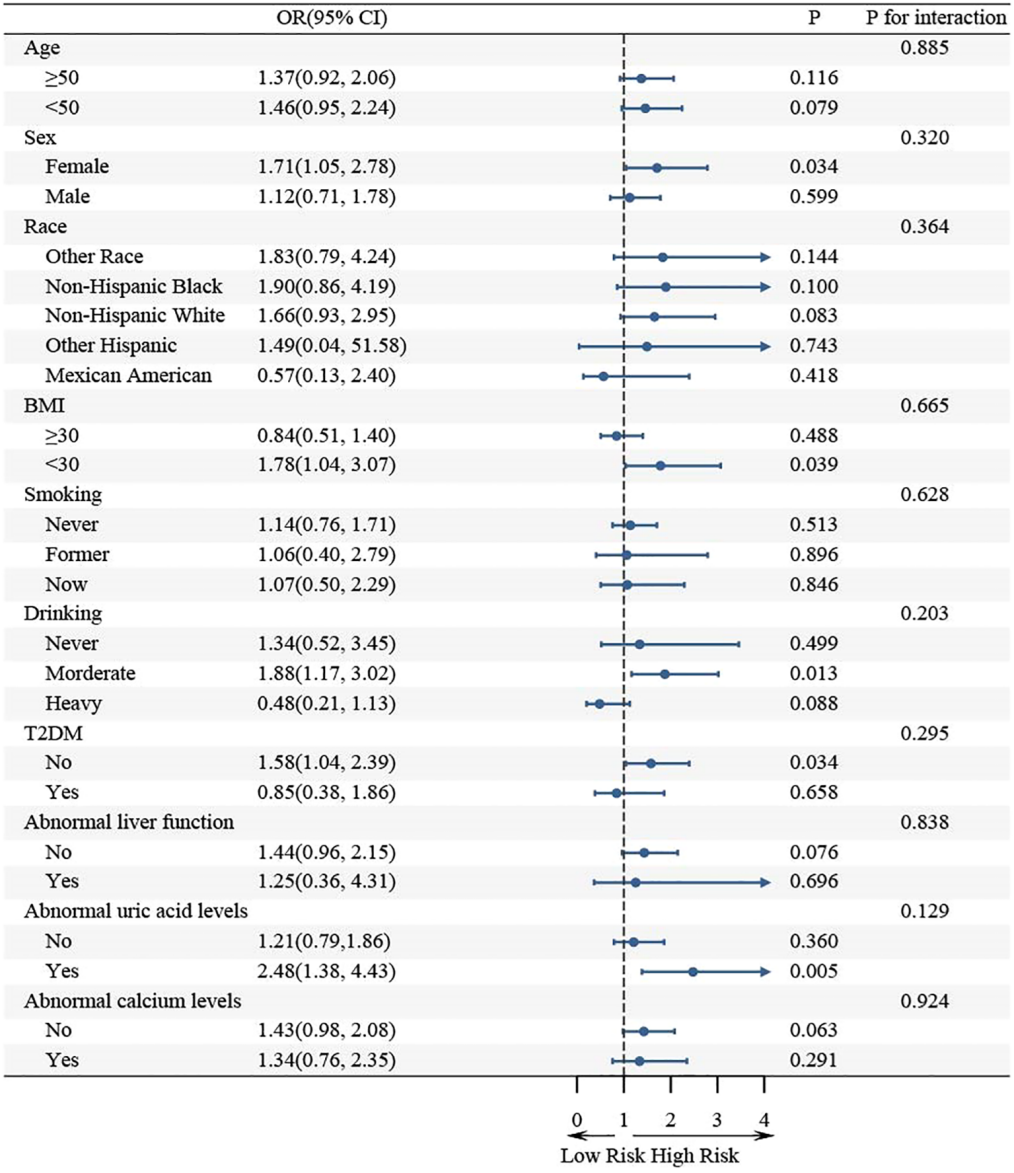
Subgroup analysis of the association between WHR and KS in hypertensive patients.

Further subgroup analysis revealed that the association between WHR and KS in hypertensive populations was not consistent across all subgroups (**Figure 4**). WHR was significantly associated with KS in the subgroup of females, BMI< 30, moderate drinking, no T2DM, and abnormal uric acid metabolism (*p*< 0.05). The association between WHR and KS remained consistent in the same direction in the remaining subgroups. Moreover, interaction tests indicated that the association between WHR and KS was not statistically different between the subgroups, suggesting that factors such as age, sex, race, BMI, smoking, drinking, T2DM, and metabolic abnormality had no statistically significant influence (*p* for interaction > 0.05).

### Sensitivity analysis

To validate the association between WHR and KS in hypertensive patients, we further adjusted the NHANES survey period based on Model 2. The results showed that the association between WHR and KS remained significant (OR = 1.63, 95% CI: 1.13, 2.35). When analyzing the WHR quartiles, patients in Q4 had a 3.45 times higher risk of KS than patients in Q1 (OR = 3.45, 95% CI: 1.32, 9.02) (**Table 4**).

**Table 4:** Sensitivity analysis for further adjustments to the survey cycle.

Parameter	OR (95%CI)	*p*
WHR^a^	1.63(1.13,2.35)	0.014
WHR quartiles		
Q1	ref	
Q2	1.08(0.46,2.54)	0.846
Q3	1.57(0.86,2.86)	0.126
Q4	3.45(1.30,9.14)	0.017
*p* for trend	0.009	

a: 1 standard deviation (SD) WHR was established in association with KS.

The model was adjusted for age, sex, race, PIR, BMI, smoking, drinking, T2DM, glucose, uric acid, calcium, ALT, AST, dietary energy, dietary protein, eGFR, physical activity, hyperlipidemia, and survey cycle.

After excluding patients with abnormal renal function, we found that there was still a significant positive association between WHR and KS (OR = 1.63, 95% CI: 1.13, 2.35) and that participants in the Q4 group had a 2.45-fold increased risk of KS compared with the Q1 group (OR = 3.45, 95% CI: 1.30, 9.14) (**Table 5**).

**Table 5:** Association between WHR and KS in hypertensive patients, excluding patients with renal dysfunction.

Parameter	Crude model		Model 1		Model 2	
	OR (95%CI)	*p*	OR (95%CI)	*p*	OR (95%CI)	*p*
WHR^a^	1.41(1.14,1.74)	0.002	1.39(1.04,1.88)	0.030	1.63(1.13,2.35)	0.014
WHR quartiles						
Q1	ref		ref		ref	
Q2	1.27(0.68,2.39)	0.448	1.23(0.69,2.19)	0.463	1.08(0.46,2.54)	0.846
Q3	1.77(0.92,3.40)	0.084	1.72(0.89,3.31)	0.102	1.57(0.86,2.86)	0.126
Q4	2.64(1.32,5.28)	0.007	2.61(1.12,6.10)	0.028	3.45(1.30,9.14)	0.017
*p* for trend		0.004		0.029		0.009

a: 1 standard deviation (SD) WHR was established in association with KS.

Crude model: no adjusted.

Model 1: adjusted for age, sex, race, and PIR.

Model 2: adjusted for age, sex, race, PIR, BMI, smoking, drinking, T2DM, glucose, uric acid, calcium, ALT, AST, dietary energy, dietary protein, eGFR, physical activity, and hyperlipidemia.

## Discussion

In this study, we investigated whether WHR might be associated with the risk of KS in hypertensive individuals. According to our findings, after controlling for confounding variables, there exists a direct and linear association between higher WHR and an increased incidence of KS among hypertensive patients. Furthermore, the association persists across a variety of subgroups within the population, demonstrating its validity.

The association between KS and hypertension is indeed bidirectional. On one hand, individuals with KS have a significantly elevated likelihood of developing hypertension compared to those without KS. Multiple studies conducted over the past two decades have consistently demonstrated a link between KS and increased hypertension risk. Notably, overweight women who develop KS exhibit a notably higher risk of hypertension (28). Furthermore, a secondary analysis of the Olivetti prospective study revealed that men with a history of KS had a 96% increased risk of developing hypertension during follow-up (29). In recent years, researchers have sought to account for potential confounding factors in examining the association between KS and hypertension. For instance, Kittanamongkolchai et al. performed a prospective study involving a baseline non-hypertensive population, aiming to investigate the association between incident KS and the incidence of hypertension. Involving 3030 participants and a follow-up period of 7.8 years, the study revealed that the first symptomatic KS was linked to a 50% increased risk of developing hypertension (30). On the other hand, hypertension itself significantly heightens the risk of KS formation. Khalili et al. discovered that hypertension, independent of potential confounders, serves as an independent risk factor for the development of KS (7). A cohort study conducted in Iran demonstrated that among individuals aged 40–70 years, hypertensive patients had a 1.43 times higher risk of developing KS compared to individuals with normal blood pressure levels (31). Collectively, these studies emphasize that hypertensive patients represent a unique population at a higher risk of KS formation.

In recent years, there has been growing research interest in exploring the role of obesity in the development of KS. Feng et al. conducted a study on adults aged ≥ 20 in the United States, finding a significant correlation between BMI and the incidence of KS. Particularly, when the BMI was< 28 kg/m^2^, this positive correlation was even more pronounced (32). Additionally, Mao et al. confirmed that populations with a BMI ≥ 30 exhibited a 51.4% higher risk of KS. Studies investigating the impact of visceral adiposity index (VAI) on KS occurrence have also yielded insightful findings (33). Liang et al. observed that participants in the highest VAI group had a 48% increased risk of KS (34). Similarly, Lin et al. reported an association between weight-adjusted waist circumference index and KS risk (35). Moreover, a longitudinal cohort study further confirmed the association between various obesity indicators, including BMI and waist circumference, and the development of KS (10). These studies emphasize the importance of obesity as a predictor of KS risk, which is consistent with our results. Unlike many studies examining the general population, our study focused specifically on hypertensive individuals. This targeted approach allowed us to better understand the unique risk factors and underlying mechanisms of KS in this high-risk population. By adjusting for multiple confounders, we provide a more precise estimate of the association between WHR and KS in hypertensive individuals. In addition, our study compares the predictive role of different obesity-related markers on the risk of KS in hypertensive patients to enhance the targeted management of this particular population.

WHR has the following advantages over other obesity-related indicators. A British study found that, independently of WHR, each standard deviation increase in BMI was associated with a 21% increase in the risk of KS, and independently of BMI, each standard deviation increase in WHR was associated with a 24% increase in the risk of KS (9). This suggests that WHR may be a more sensitive indicator of centralized obesity, which is particularly important in the assessment of KS risk. Furthermore, WHR is a relatively simple and non-invasive indicator that can be easily calculated by measuring waist and hip circumference. Unlike the VAI, which requires multiple biochemical parameters (e.g., triglycerides, HDL cholesterol) and is more complex to calculate, the WHR can be easily assessed in a clinical setting without the need for special equipment or laboratory tests (36). This simplicity makes the WHR a practical tool for screening and monitoring KS risk in hypertensive patients. In contrast, BMI does not differentiate fat distribution and may underestimate risk in individuals with normal BMI but central obesity. The VAI, although more comprehensive, may be less practical for routine clinical use because of its complexity.

While we observed an association between WHR and KS likelihood, the underlying mechanisms remain unclear. WHR is a crucial indicator of abdominal obesity. We considered the following potential mechanisms. First, central obesity is closely associated with insulin resistance. Insulin resistance can lead to hyperinsulinemia, which in turn promotes renal reabsorption of sodium and calcium, thereby increasing urinary calcium excretion, a metabolic disorder that is particularly important in hypertensive patients, who are often comorbid with the metabolic syndrome (37–39). In addition, obesity has been associated with a chronic, low-grade inflammation characterized by tumor necrosis factor-α (TNF-α), elevated levels of pro-inflammatory cytokines such as interleukin-6 (IL-6) and C-reactive protein (CRP) (40). These cytokines affect renal hemodynamics and tubular function and may lead to increased stone formation (41). In hypertensive patients, the combination of central obesity and hypertension may exacerbate the inflammatory process, further increasing the risk of KS. Finally, central obesity is usually accompanied by dyslipidemia as evidenced by elevated triglycerides and decreased levels of HDL cholesterol (42). Dyslipidemia can lead to the formation of cholesterol stones, especially in the setting of hypertension (43). The combination of central obesity and hypertension may lead to an increased risk of KS.

There are some subgroups in which CIs spanning 1 may indicate no significant association between WHR and KS. However, this does not necessarily imply a true no effect, but rather a lack of statistical efficacy that may be due to insufficient sample size. For example, in the male subgroup, although the confidence interval spanned 1, the ratio of ratios (OR = 1.52) still indicated a trend toward a positive association between WHR and KS. Likewise, in the BMI ≥ 30 subgroup, an OR of 1.45 likewise points toward a positive association, but the wide CI renders the estimate non-significant. To further validate the effects in these subgroups, we suggest that more detailed analyses be conducted for these specific subgroups in future large studies. This will help determine the true relationship between WHR and KS in these subgroups and provide more reliable conclusions.

Central obesity is closely associated with metabolic syndrome, including hypertension, hyperglycemia, and dyslipidemia. These metabolic disorders are key factors in KS. By focusing on WHR, our study provides insights for endocrine and metabolic research on the specific role of central obesity in this context. By identifying central obesity through WHR, clinicians can better target interventions to reduce the risk of KS in this high-risk population.

Our study is based on a representative US population. After carefully considering potential confounding factors, we identified an association between WHR and an increased likelihood of KS occurrence in the hypertensive population. However, our study also has a few limitations. Firstly, as a cross-sectional study, we cannot establish a causal association between WHR and KS development. Future cohort studies or randomized controlled trials are necessary to explore the causal linkage between WHR and KS. Secondly, although we adjusted for common confounding factors in our study, there is a possibility that other potential factors were not accounted for, and thus, we should interpret the results and draw conclusions with caution. Thirdly, the NHANES database does not provide information on the recurrence of KS, which is an important aspect of KS. Recurrence rates are critical for understanding the long-term impact of risk factors such as hypertension and central obesity on KS. Future studies should consider incorporating data on KS recurrence to provide a more comprehensive understanding of the disease. Lastly, since our study subjects were exclusively from the US population, further research is needed to determine whether these findings can be generalized to other populations in different regions.

## Conclusions

In the population with hypertension, our study found a significant association between WHR and the likelihood of KS. This association remained significant even after adjusting for potential confounding factors. Specifically, we observed an independent and linear association, indicating that higher WHR is associated with an increased likelihood of developing KS. These findings highlight the significance of managing abdominal fat as a means of controlling the occurrence of KS in individuals with hypertension.

## Data Availability

The raw data supporting the conclusions of this article will be made available by the authors, without undue reservation.
