# Supplementary material for: Association of waist-to-hip ratio with risk of kidney stones in hypertensive population: a population-based cross-sectional study
Source: Front Endocrinol (Lausanne). 2025 Sep 23;16:1448137. doi: 10.3389/fendo.2025.1448137 (PMC12501787; doi:10.3389/fendo.2025.1448137)
Supplement: Supplementary file 1 [file Table1.docx]

**Supplementary Material**

**Supplementary table 1. Association between obesity-related indicators (WC, BMI, VAI, and TyG)and KS in hypertensive patients**

| **Supplementary table 1.** Association between obesity-related indicators (WC, BMI, VAI, and TyG)and KS in hypertensive patients | | | | | | |
| --- | --- | --- | --- | --- | --- | --- |
|  | Crude model | | Model 1 | | Model 2 | |
|  | OR (95%CI) | *p* | OR (95%CI) | *p* | OR (95%CI) | *p* |
| WC^a^ | 1.37(1.05,1.69) | 0.021 | 1.35(1.03,1.78) | 0.031 | 1.34(1.02,1.66) | 0.036 |
| WC quartiles |  |  |  |  |  |  |
| Q1 | ref |  | ref |  | ref |  |
| Q2 | 1.12(0.77,2.19) | 0.052 | 1.10(1.02,2.65) | 0.040 | 1.07(0.95,2.21) | 0.073 |
| Q3 | 1.52(0.86,2.61) | 0.067 | 1.55(0.95,2.83) | 0.121 | 1.49(0.94,2.85) | 0.091 |
| Q4 | 2.56(1.84,3.88) | 0.015 | 2.44(2.04,3.79) | 0.027 | 2.10(1.41,3.98) | 0.011 |
| BMI^a^ | 1.23(1.03,1.48) | 0.021 | 1.22(1.03,1.47) | 0.040 | 1.20(1.01,1.56) | 0.045 |
| BMI quartiles |  |  |  |  |  |  |
| Q1 | ref |  | ref |  | ref |  |
| Q2 | 0.97(0.57,1.38) | 0.931 | 0.88(0.42,1.34) | 0.723 | 0.81(0.42,1.47) | 0.511 |
| Q3 | 1.20(0.90,1.58) | 0.093 | 1.14(0.87,1.46) | 0.110 | 1.10(0.85,1.40) | 0.131 |
| Q4 | 1.82(1.45,2.48) | 0.008 | 1.80(1.43,2.49) | 0.007 | 1.65(1.35,2.21) | 0.016 |
| VAI^a^ | 1.27(1.03,1.86) | 0.009 | 1.27(1.04,1.86) | 0.013 | 1.11(1.01,1.71) | 0.020 |
| VAI quartiles |  |  |  |  |  |  |
| Q1 | ref |  | ref |  | ref |  |
| Q2 | 0.97(0.47,1.88) | 0.093 | 0.91(0.46,1.83) | 0.079 | 0.83(0.41,1.65) | 0.056 |
| Q3 | 1.38(0.92,2.36) | 0.074 | 1.40(0.92,2.44) | 0.078 | 1.21(0.81,2.14) | 0.054 |
| Q4 | 1.76(1.44,2.31) | 0.002 | 1.72(1.40,2.29) | 0.006 | 1.60(1.27,2.33) | 0.019 |
| TyG^a^ | 1.30(1.12,1.66) | 0.005 | 1.25(1.07,1.59) | 0.007 | 1.27(1.06,1.68) | 0.008 |
| TyG quartiles |  |  |  |  |  |  |
| Q1 | ref |  | ref |  | ref |  |
| Q2 | 1.14(0.62,1.73) | 0.171 | 1.13(0.79,1.89) | 0.230 | 1.12(0.80,1.70) | 0.205 |
| Q3 | 1.32(0.73,2.05) | 0.880 | 1.23(0.75,1.66) | 0.763 | 1.22(0.66,1.78) | 0.515 |
| Q4 | 1.54(1.13,2.86) | 0.004 | 1.69(1.33,2.31) | 0.007 | 1.65(1.28,2.26) | 0.009 |
| a: 1 SD indicators markers was established in association with KS | | | | | | |
| Crude model: no adjusted; | | | | | | |
| Model 1: adjusted for age, sex, race, and PIR; | | | | | | |
| Model 2: adjusted for age, sex, race, PIR, BMI, smoking, drinking, T2DM, glucose, uric acid, calcium, ALT, AST, dietary energy, dietary protein, eGFR, physical activity, and hyperlipidemia. | | | | | | |
